# High-Dose Zinc Supplementation Therapy Does Not Improve Survival Rates in Severe Trauma Patients: A Single-Center Retrospective Observational Study

**DOI:** 10.3390/nu18030541

**Published:** 2026-02-06

**Authors:** Ryota Tsushima, Takaaki Maruhashi, Muneyoshi Kim, Yasushi Asari

**Affiliations:** Department of Emergency and Critical Care Medicine, Kitasato University School of Medicine, 1-15-1 Kitasato, Minami-ku, Sagamihara 252-0375, Japan; maru119@kitasato-u.ac.jp (T.M.); kin.muneyoshi@kitasato-u.ac.jp (M.K.); ya119@kitasato-u.ac.jp (Y.A.)

**Keywords:** severe trauma, zinc supplementation, mechanical ventilation, survival rate, intensive care, critical care nutrition, 30-day mortality, retrospective observational study

## Abstract

**Background/Objectives:** Hypozincemia associated with severe trauma contributes to immune dysfunction and poor prognosis; however, the clinical utility and optimal dosage of zinc supplementation remain unclear. In particular, it is unclear whether high-dose administration exceeding standard recommendations improves prognosis. Thus, we aimed to verify this in patients with severe trauma requiring mechanical ventilation. **Methods:** This single-center retrospective observational study included patients with severe trauma (Injury Severity Score [ISS] > 15) requiring mechanical ventilation admitted to our emergency intensive care unit (ICU) between April 2015 and March 2023. Patients were classified into three groups based on their mean daily zinc supplementation dose: low (<15 mg), medium (15–50 mg), and high (>50 mg). The primary outcome was the 30-day survival rate. Secondary outcomes included the 90-day survival rate, length of ICU stay, and hospital-acquired pneumonia. Multivariable regression evaluated the association between high-dose zinc supplementation and clinical outcomes after adjusting for confounding factors. **Results:** Of 196 patients, 86, 16, and 94 were in the low-, medium-, and high-dose groups, respectively. The high-dose group had significantly poorer nutritional status and lower serum zinc levels, whereas no significant differences were observed in severity scores or study outcomes. High-dose zinc supplementation showed no significant association with improved 30-day survival in adjusted analyses. **Conclusions:** In patients with severe trauma requiring mechanical ventilation, high-dose zinc supplementation did not improve 30-day survival or other clinical outcomes compared with standard doses. These results do not support the use of high-dose zinc supplementation for severe trauma.

## 1. Introduction

Severe trauma triggers life-threatening critical conditions, including excessive systemic inflammatory responses, oxidative stress, and subsequent immunoparalysis initiated by extensive tissue injury and ischemia–reperfusion injury [1]. Controlling this complex series of biological reactions to save lives remains a significant challenge in intensive care.

Following iron, zinc is the second most plentiful essential trace element found in the human body, playing roles in the maintenance of immune function [2,3], protection against oxidative stress [4,5,6], and wound healing [7]. Under conditions of high invasiveness, while the biological demand for zinc increases rapidly, patients develop hypozincemia owing to absorption disorders [8], alterations in internal distribution [9], and increased external loss [10]. Consequently, defense and repair mechanisms break down, contributing to secondary infections, multiple organ failure, and increased mortality rates. In observational studies targeting patients with severe trauma, those with hypozincemia at two weeks post-admission showed significantly worse outcomes, including longer intensive care unit (ICU) stays, prolonged mechanical ventilation, higher infection complication rates, and increased blood culture positivity rates, compared with zinc-sufficient groups [11]. Therefore, zinc supplementation in patients with severe trauma can be considered a pathophysiologically rational therapeutic intervention. Historically, Young et al. [12] demonstrated that zinc supplementation, including intravenous administration to rapidly normalize serum levels, was associated with improved neurological recovery rates and visceral protein levels in patients with severe closed head injury. Subsequently, other studies have also reported shortened burn healing times in patients with severe burns [13] and improved neurological outcomes in patients with severe head trauma [14] in zinc-supplemented groups.

Conversely, a recent meta-analysis focusing on trauma patients (barring burn cases) revealed that while zinc supplementation did not significantly improve primary outcomes like mortality or hospitalization duration, it did lead to a significant decrease in pneumonia rates [15]. However, evidence supporting the clinical efficacy of zinc supplementation remains insufficient. Another unresolved issue is that the optimal clinical dosage of zinc has not yet been established. While European Society for Clinical Nutrition and Metabolism (ESPEN) guidelines recommend at least 10 mg per 1500 kcal per day for enteral nutrition and 3–5 mg per day for parenteral nutrition [16], major intensive care societies in Japan and the US (Society of Critical Care Medicine [SCCM], American Society for Parenteral and Enteral Nutrition [ASPEN], and Japanese Society of Intensive Care Medicine [JSICM]) currently provide no clear criteria. The lack of consistent efficacy in previous studies may be because the dosages were insufficient to exert clinical effects.

Our previous institutional research on septic patients failed to demonstrate the clinical utility of high-dose zinc supplementation [17]. Nevertheless, we reasoned that the clinical impact of zinc might be more pronounced in severe trauma than in sepsis. Unlike the primarily inflammatory nature of sepsis, traumatic injury involves massive structural tissue damage that necessitates accelerated DNA synthesis and protein production for repair—processes in which zinc serves as a fundamental cofactor. Based on this rationale, we hypothesized that high-dose zinc supplementation, by meeting the heightened demand for tissue regeneration and immune stabilization, could improve the prognosis of severe trauma. The purpose of this study was to verify the impact of high-dose zinc supplementation on clinical outcomes and to examine the optimal dosage using retrospective cohort data of patients with severe trauma at our institution.

## 2. Materials and Methods

### 2.1. Study Design and Setting

A single-center, retrospective observational study was conducted to evaluate severe trauma patients admitted to our institution between 1 April 2015, and 31 March 2023. The study design and data collection protocols were adapted from a previous retrospective study conducted at our institution [17]. Severe trauma was defined as cases requiring mechanical ventilation with an Injury Severity Score (ISS) > 15, calculated according to the AIS-90 Update 98. The exclusion criteria included the presence of non-severe trauma with an ISS ≤ 15, difficulty administering enteral nutrition, and death within 24 h of ICU admission.

This study was conducted at a regional core hospital with approximately 1200 beds, including a 20-bed emergency and critical care intensive care unit (EICU) staffed by intensive care specialists. Nutritional therapy adhered to ESPEN guidelines, commencing with small-volume enteral feeding via a nasogastric tube within 48 h of admission. Target caloric intake was determined using indirect calorimetry. A strategy of permissive underfeeding was adopted initially, with intake titrated to the full caloric target over one week; the protein intake goal was set at ≥1.2 g/kg. Additionally, the standard protocol included the administration of 125 mg of the trace element preparation V CRESC^®^ (Nutri Co., Ltd., Yokkaichi, Japan) from the initiation of enteral nutrition. Serum zinc levels and other nutritional markers were assessed at baseline and weekly thereafter. Any zinc supplementation beyond the standard V CRESC^®^ dose was prescribed at the attending physician’s discretion based on laboratory findings.

### 2.2. Data Collection and Study Outcomes

First, mechanically ventilated patients were identified from the ICU admission database. From this population, we selected trauma patients presenting with an Injury Severity Score (ISS) > 15. We extracted the following variables from electronic medical records: demographics (age, sex), anthropometrics (body weight, body mass index [BMI]), comorbidities, nutritional status (Controlling Nutritional Status [CONUT] score), and severity indices at admission (Acute Physiology and Chronic Health Evaluation [APACHE] II score, ISS). Clinical outcomes, including duration of mechanical ventilation, requirement for renal replacement therapy, ICU length of stay, discharge outcome, and hospital-acquired pneumonia (defined as onset ≥ 48 h after admission), were also recorded. Laboratory data—specifically white blood cell count, lymphocyte count, platelet count, total bilirubin, creatinine, total protein, albumin, total cholesterol, and C-reactive protein—were collected at admission and at weeks 1, 2, and 3.

Consistent with the methodology established in our previous research [16], zinc supplementation dosages were standardized to elemental zinc content: 12 mg for V CRESC^®^, 17 mg for polaprezinc, 70 mg for zinc acetate hydrate, and 134.2 mg for zinc sulfate hydrate. These preparations were administered via oral or enteral routes; intravenous zinc was excluded due to its negligible content.

Patients were stratified into three cohorts based on mean daily zinc dosage: <15 mg, 15–50 mg, and >50 mg. The primary endpoint was the 30-day survival rate. Secondary endpoints included 90-day survival, ICU length of stay, incidence of hospital-acquired pneumonia, and temporal changes in serum zinc concentrations from weeks 1 to 3. Pneumonia was diagnosed by the attending physicians through a comprehensive evaluation of imaging, clinical, biochemical, and microbiological findings. The criteria included the presence of new or progressive pulmonary infiltrates on chest imaging, accompanied by clinical signs (e.g., fever, purulent secretions), biochemical evidence of inflammation (e.g., elevated white blood cell count or C-reactive protein levels), and/or microbiological evidence from respiratory tract specimens (e.g., Gram staining or cultures).

### 2.3. Statistical Analysis

For continuous variables, data were presented as medians and interquartile ranges; differences were assessed using the Mann–Whitney *U* test or the Kruskal–Wallis test, as appropriate. Binary variables were described as percentages and compared using Fisher’s exact test. We utilized the Kaplan–Meier method to evaluate 30-day and 90-day survival rates among the three groups. Hazard ratio (HR) and 95% confidence interval (CI) values for 30-day mortality were estimated using a univariate Cox proportional hazards model, with the <15 mg group as the reference. Multivariate logistic regression analysis using the forced-entry method was also performed to identify associations between zinc supplementation dosage and key outcomes (30-day survival, 90-day survival, and pneumonia onset). As a sensitivity analysis, the 15–50 mg group was excluded, and a direct comparison was made between the <15 mg group and the >50 mg group using the log-rank test, Cox proportional hazards models, and multivariate logistic regression. Statistical significance was defined as *p* < 0.05. All analyses were conducted using IBM SPSS Statistics ver. 26 (IBM Corp., Armonk, NY, USA).

### 2.4. Ethics

In adherence to the Declaration of Helsinki, this study was conducted with the approval of the Kitasato University Hospital Institutional Review Board (Approval Number: B25-064). Due to the retrospective nature of the analysis, the board waived the need for individual informed consent, allowing for an opt-out approach instead.

## 3. Results

**Figure 1:**
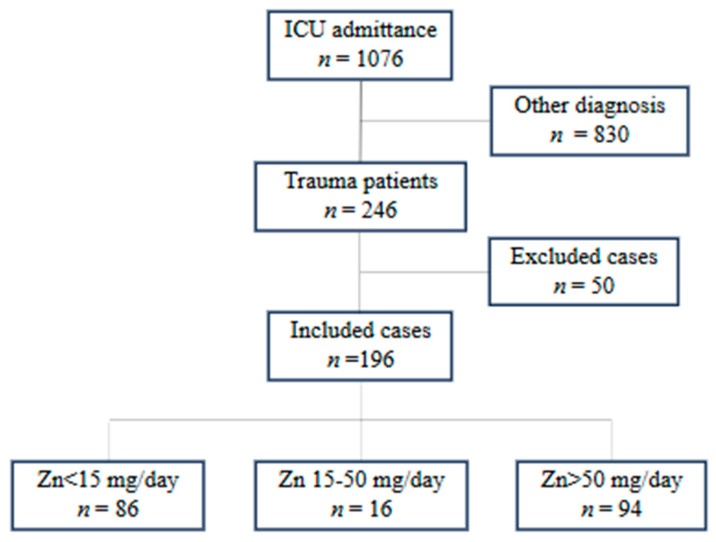
Flowchart of patient selection. Of 1076 patients who received mechanical ventilation in the ICU, 246 trauma patients were screened. Fifty patients with an ISS ≤ 15 were excluded. The remaining 196 patients were included in the analysis and stratified into three groups based on mean daily zinc dosage: <15 mg/day (*n* = 86), 15–50 mg/day (*n* = 16), and >50 mg/day (*n* = 94).

Of 1076 patients who received mechanical ventilation in the ICU, 246 presented with trauma. Among them, 50 with an ISS ≤ 15 met the exclusion criteria, leaving 196 patients for analysis. Participants were subsequently stratified into three groups according to zinc supplementation dosage, as detailed in Figure 1: the <15 mg group (*n* = 86), the 15–50 mg group (*n* = 16), and the >50 mg group (*n* = 94). The specific types and combinations of zinc formulations used to achieve these dosages are summarized in Table 1. All patients in the <15 mg group (100.0%) received zinc solely through a nutritional supplement (V CRESC^®^). In contrast, the 15–50 mg and >50 mg groups predominantly received medicinal zinc preparations in addition to the supplement. In the 15–50 mg group, the combination of polaprezinc and V CRESC^®^ was most frequent (93.8%), while the >50 mg group predominantly used zinc sulfate (70.2%) or zinc acetate (24.5%) in combination with V CRESC^®^.

Table 2 summarizes the baseline characteristics of the study population. The three groups were comparable regarding demographics and severity; specifically, no statistically significant differences were detected in age, sex, body weight, BMI, ISS, or APACHE-II scores.

Only the CONUT score differed significantly (7.0 (5.0–8.0) vs. 7.0 (5.0–8.0) vs. 8.0 (7.0–10.0); *p* = 0.048). With the exception of albumin and zinc, baseline laboratory parameters—including white blood cell count, lymphocyte count, platelet count, total bilirubin, creatinine, total protein, total cholesterol, and C-reactive protein—showed no significant intergroup differences. Significant disparities were noted in albumin levels (2.75 [2.30–3.10] vs. 2.70 [2.40–2.80] vs. 2.30 [2.05–2.70]; *p* = 0.01) and zinc levels (46.0 [37.0–59.0] vs. 41.0 [30.0–47.0] vs. 34.0 [27.0–42.5]; *p* < 0.01).

**Figure 2:**
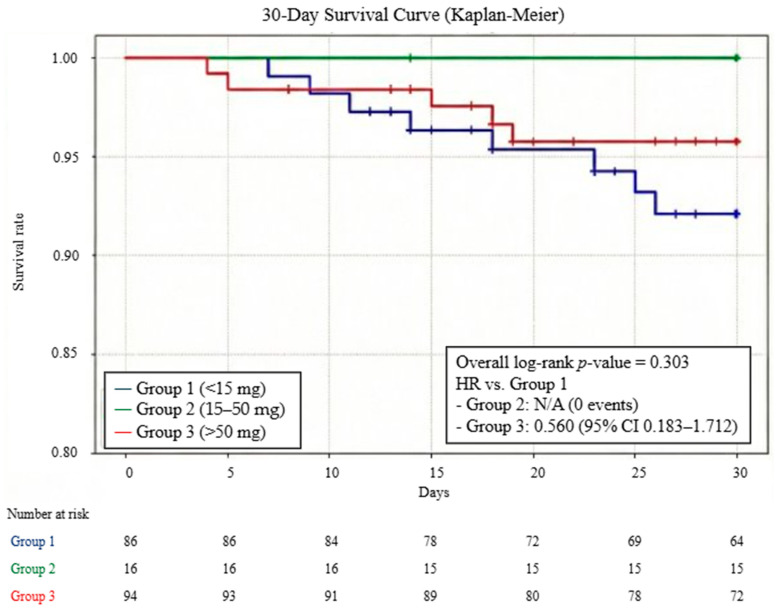
Kaplan–Meier estimates of 30-day survival according to mean daily zinc dosage. Patients were stratified into three cohorts based on mean daily zinc dosage: <15 mg (Group 1), 15–50 mg (Group 2), and >50 mg (Group 3). The overall difference in survival was compared using the log-rank test. Hazard ratio (HR) and 95% confidence intervals (CI) were calculated using a univariate Cox proportional hazards model with Group 1 as the reference. The HR for Group 2 was not calculable due to the absence of mortality events.

Regarding the primary endpoint, the 30-day survival rate showed no statistically significant difference among the three groups (<15 mg: 90.7% [95% CI 84.5–96.9%], 15–50 mg: 100%, and >50 mg: 94.7% [95% CI 90.1–99.3%]; overall log-rank *p* = 0.303). The HR for the >50 mg group compared with the <15 mg group was 0.560 (95% CI 0.183–1.712; *p* = 0.31). For the 15–50 mg group, the HR was not calculable due to the absence of mortality events during the study period (Figure 2). Furthermore, a sensitivity analysis excluding the intermediate-dose group (15–50 mg) was conducted to compare the low-dose (<15 mg) and high-dose (>50 mg) groups directly. This analysis revealed no significant difference in 30-day survival between the two groups (HR 0.576; 95% CI 0.176–1.889; log-rank *p* = 0.356).

Secondary endpoints also demonstrated no statistically significant variations among the groups: 90-day survival rate (88.3 [95% CI 81.0–95.6] vs. 85.6 [95% CI 66.9–100.0] vs. 86.2 [95% CI 77.9–94.5]; *p* = 0.97); ICU length of stay (14 [10–18] vs. 16 [10–21] vs. 16 [11–19]; *p* = 0.35); hospital-acquired pneumonia (54.7 [95% CI 44.2–64.8] vs. 31.3 [95% CI 14.2–55.6] vs. 42.6 [95% CI 33.0–52.7]). Similarly, temporal trends in blood zinc concentrations did not differ significantly between the groups at weeks 2 and 3 (*p* = 0.37 and 0.95, respectively) (Table 3).

Patient backgrounds and clinical data were compared between the 30-day survivor (*n* = 158) and non-survivor (*n* = 11) groups (Table 4). Univariate analysis revealed a significantly higher percentage of males in the non-survivor group compared to the survivor group (73.8% vs. 38.5%, *p* = 0.011). No significant differences were observed between the two groups regarding age, BMI, severity scores (ISS and APACHE II), nutritional status (CONUT score), or renal replacement therapy.

To examine the effect of zinc supplementation on 30-day survival, a multivariate logistic regression analysis was performed (Table 5). Because no deaths occurred in the second group (15–50 mg/day), it was combined with the first group (<15 mg/day) to form the “≤50 mg/day group” for statistical stability and then compared with the high-dose group (>50 mg/day). After adjusting for age and CONUT score, high-dose zinc supplementation showed no statistically significant association with 30-day survival compared with the control group (adjusted odds ratio [aOR] 0.89; 95% CI 0.25–3.24; *p* = 0.86). Consistent with the primary analysis, multivariate logistic regression excluding the second group (15–50 mg/day) revealed that high-dose zinc supplementation was not significantly associated with 30-day mortality when compared to the low-dose group (aOR 0.73; 95% CI 0.20–2.67; *p* = 0.64).

Using a similar multivariate analysis model, 90-day survival and the onset of hospital-acquired pneumonia were examined. High-dose zinc supplementation showed no significant association with 90-day survival (adjusted odds ratio 1.11; 95% CI 0.40–3.05; *p* = 0.85), while age was a significant risk factor (*p* = 0.02) (Table 6). The same findings were observed for hospital-acquired pneumonia. Similarly, (adjusted odds ratio 0.64; 95% CI 0.34–1.22; *p* = 0.18), with age remaining a significant risk factor (*p* < 0.01) (Table 7).

## 4. Discussion

In this study, we examined differences in clinical outcomes based on zinc supplementation doses in patients with severe trauma and found that increasing zinc supplementation did not improve blood zinc concentrations or clinical outcomes (survival rate, length of ICU stays, or incidence of hospital-acquired pneumonia) in the acute phase.

The lack of effect on zinc concentrations in the 2nd and 3rd weeks despite high-dose supplementation may be due to changes in zinc pharmacokinetics in the acute phase. Under normal conditions, dietary zinc is taken up into intestinal epithelial cells via the ZIP4 transporter in the small intestine and then sent to the portal circulation via the ZnT1 transporter, absorbed into the body, and finally excreted via feces and gastrointestinal secretions. Zinc in the body is distributed as follows: 85–90% in skeletal muscle and bone, 4.2% in the skin, 3.4% in the liver, and in several amounts in other organs, including the blood. Clinical studies on zinc pharmacokinetics in patients with trauma and burns have indicated zinc redistribution, increased urinary zinc excretion [18], and zinc loss from exudates [19]. Consistently, basic experiments in rat models of hemorrhagic shock have shown a marked decrease in zinc albumin-binding capacity, which is considered a biological mechanism of redistributing zinc to injured tissues by increasing free zinc [20]. Furthermore, in sepsis, plasma zinc has been reported to redistribute to organs owing to changes in zinc transporter expression. In this inflammatory context, large amounts of inflammatory cytokines such as IL-6 and IL-8 are produced, serving as signals that specifically upregulate the expression of the zinc transporter SLC39A8 in immune cells. This increase promotes active uptake of zinc from plasma into cells, resulting in decreased blood zinc concentration [9]. Similarly, in trauma, similar to sepsis, damage-associated molecular patterns generated by tissue injury and ischemia–reperfusion are recognized by Toll-like receptors, further inducing systemic inflammation; this is known to cause multiple organ injury and affect outcomes. Therefore, changes in zinc pharmacokinetics during the acute phase of sepsis likely occur similarly in trauma patients, suggesting that differences in zinc supplementation amounts may not lead to changes in serum zinc concentrations. Consequently, the failure to increase serum zinc concentrations may be the primary factor for the lack of clinical improvement observed in this study.

An alternative interpretation of our results is that the potential therapeutic benefits of high-dose zinc were masked by the significantly poorer baseline nutritional status and higher severity in the high-dose group. Patients in the >50 mg group exhibited significantly lower baseline serum albumin and zinc levels, along with numerically higher ISS, compared to other groups. This clinical imbalance suggests “confounding by severity,” where patients with clinically worse conditions were more likely to receive high doses. Therefore, the lack of a significant difference in survival rates might not necessarily indicate a lack of efficacy; instead, it could suggest that high-dose zinc supplementation helped “neutralize” the inherently higher mortality risk in these severely compromised patients, bringing their outcomes closer to those of the less severely injured patients in the low-dose group.

Importantly, the efficacy of zinc supplementation may vary significantly depending on the type of trauma, overall severity, and the timing of administration. Regarding the type of trauma, diverse injury patterns, such as blunt versus penetrating trauma, create heterogeneous metabolic demands. For instance, patients with extensive soft tissue injuries or major burns may have considerably different zinc requirements than those with isolated head trauma; however, these patterns were not separately analyzed in this study. In terms of overall severity, a standardized dosing approach may fail to address the varying degrees of systemic inflammation and zinc redistribution across different severity levels. Furthermore, the timing of administration is a critical factor for clinical efficacy. In patients with severe head trauma, zinc demand has been reported to peak within 24 h of injury [18], and it is postulated that a similar trend occurs in severe trauma in general. In the present study, however, zinc was administered orally, making supplementation within the first 24 h of injury unfeasible. This suggests that the “window of opportunity” for effective supplementation may have been missed in our cohort. Consequently, future research should consider the use of intravenous administration to facilitate earlier intervention, along with more tailored strategies for diverse trauma populations.

In addition to these pharmacokinetic and methodological considerations, high-dose zinc supplementation may have failed to improve clinical outcomes owing to concurrent potential adverse events caused by copper deficiency. Intestinal mucosal cells express metallothionein, a low-molecular-weight protein that binds strongly to metal ions and neutralizes the toxicity of heavy metals (such as Zn, Cu, and Cd) intracellularly [6]. Excessive zinc in the intestinal tract leads to the activation of MTF-1, a zinc finger protein, which migrates into the nucleus of intestinal mucosal cells and binds to metal response elements, the promoter region of the metallothionein gene. Consequently, transcription of the metallothionein gene is promoted, and metallothionein protein expression is markedly upregulated [21]. The binding affinity of this metallothionein protein is significantly higher for copper than for zinc, causing copper to be preferentially captured and its absorption from the intestinal tract to decrease [22]. Notably, copper serves a pivotal function in preserving physiological equilibrium across diverse organ systems, including the hematologic system [23], nervous system [24], and skin and connective tissues [25]. Therefore, some reports suggest that zinc supplementation should be maintained within 45 mg/day [26]. In this study, the high-dose zinc supplementation group may have had concurrent adverse events owing to copper deficiency, which might have further offset any potential benefits of zinc. However, blood copper was not measured in this study, remaining a subject for future verification. Nevertheless, this study does not negate the efficacy of zinc supplementation itself. Previous studies have suggested that the incidence of infection may be reduced with standard amounts of zinc supplementation. Given that no significant difference in clinical outcomes was observed between the <15 mg group and the high-dose zinc supplementation groups (15–50 mg group, >50 mg group), it is suggested that standard dosage (<15 mg) may be sufficient from the perspective of prognosis improvement.

This study had several limitations. First, it was a retrospective observational study. As discussed, the high-dose zinc group was inherently at a higher risk of poor outcomes due to significantly poorer baseline nutrition and higher ISS. Although we performed multivariate adjustments using three key variables (zinc supplementation, age, and CONUT score), the limited number of deaths and statistical power raise the possibility of residual confounding and an underestimation of zinc’s effects. Second, the heterogeneous nature of trauma—including varying injury patterns and the timing of zinc initiation—could not be fully accounted for. Specifically, since zinc was administered orally in our cohort, it was unfeasible to start supplementation within the first 24 h of injury, the period when zinc demand is believed to peak. This may have resulted in missing the optimal “window of opportunity” for effective intervention. Third, the chemical forms of the zinc formulations were not uniform across the study groups. Existing literature indicates that zinc bioavailability varies significantly by chemical form, and absorption rates have been reported to range from 59% to 70% depending on the specific formulation [27,28,29,30]. Such differences in absorption could be biologically meaningful. In the present study, the <15 mg group received zinc primarily through a nutritional supplement (V CRESC^®^), whereas the 15–50 mg and >50 mg groups were administered medicinal preparations, such as zinc sulfate, zinc acetate, or polaprezinc. This lack of uniformity in zinc sources may have contributed to the variability in reported outcomes and could limit the direct comparison of clinical efficacy based solely on the total elemental zinc dosage.

Several additional limitations related to outcome assessment, trace element monitoring, and generalizability should also be considered. Additionally, the retrospective nature of this study precluded the evaluation of other clinically relevant secondary endpoints, such as objective wound healing scores or longitudinal changes in infection-related biomarkers. These parameters were not consistently or systematically recorded in the clinical charts, preventing an exploratory analysis of the impact of zinc supplementation on these “soft” clinical outcomes. Moreover, blood copper concentrations were not routinely measured. Consequently, we could not evaluate the possibility that high-dose administration caused iatrogenic copper deficiency, which may have adversely affected clinical outcomes. Furthermore, although serum zinc concentrations after admission were comparable between groups, urinary zinc excretion and tissue zinc concentrations were not measured; thus, we could not evaluate zinc pharmacokinetics. In addition, this study was conducted over a relatively long period (2015–2023). While trauma care protocols, zinc administration practices, and medical equipment/infrastructure remained consistent at our institution, we cannot completely rule out the influence of temporal factors, such as subtle advances in intensive care medicine or lifestyle changes, on survival rates. Due to the limited sample size, we did not perform a year-by-year analysis, which remains a subject for future prospective studies with larger cohorts. Finally, this study was conducted at a single center. Therefore, further research with more cases across multiple centers is necessary to verify the external validity of the study results.

## 5. Conclusions

In patients with severe trauma requiring mechanical ventilation, high-dose oral zinc supplementation did not significantly improve 30-day survival or other clinical outcomes compared with standard doses. These results suggest that a standard zinc supplementation dose (less than 15 mg/day) may be sufficient for the management of such patients. However, an alternative interpretation is that the high-dose zinc supplementation helped to “neutralize” the inherently higher mortality risk in the more severely compromised patients, who were more likely to receive higher doses. Therefore, future prospective randomized controlled trials are warranted to overcome these limitations and verify the true clinical efficacy and optimal dosing of zinc in severe trauma.

## Figures and Tables

**Table 1:** Zinc formulation combinations across the study groups.

	<15 mg/day	15–50 mg/day	>50 mg/day
	(N = 86)	(N = 16)	(N = 94)
Monotherapy, No. (%)			
V CRESC^®^ alone	86 (100)	0 (0)	0 (0)
Polaprezinc alone	0 (0)	1 (6.2)	0 (0)
Zinc sulfate alone	0 (0)	0 (0)	2 (2.1)
Combination therapy, No. (%)			
Polaprezinc + V CRESC^®^	0 (0)	15 (93.8)	0 (0)
Zinc acetate + V CRESC^®^	0 (0)	0 (0)	23 (24.5)
Zinc sulfate + V CRESC^®^	0 (0)	0 (0)	66 (70.2)
Others *	0 (0)	0 (0)	3 (3.2)

* Others include combinations of polaprezinc + zinc sulfate with or without V CRESC^®^.

**Table 2:** Sample background and clinical characteristics.

	<15 mg/day	15–50 mg/day	>50 mg/day	*p*-Value
	(N = 86)	(N = 16)	(N = 94)	
Age, median [IQR], year	63.0 [42.0–78.5]	50.0 [32.0–72.0]	61.0 [42.0–73.5]	0.45
Male sex, No. (%)	60 (70)	12 (75)	68 (72)	0.88
Weight, median [IQR], kg	66.30 [55.70–76.20]	66.80 [61.30–72.60]	67.00 [58.5–74.85]	0.78
BMI, median [IQR]	23.71 [21.39–26.60]	25.08 [24.08–27.61]	24.99 [22.60–27.36]	0.98
CONUT score, median [IQR]	7.0 [5.0–8.0]	7.0 [5.0–8.0]	8.0 [7.0–10.0]	0.048
ISS, median [IQR]	26.0 [25.0–34.0]	27.0 [17.0–38.0]	32.0 [25.0–39.5]	0.18
APACHE II score, median [IQR]	26.0 [22.0–30.0]	26.0 [24.0–30.0]	26.0 [21.0–31.0]	0.99
Laboratory findings upon arrival, median [IQR]				
White blood cell count, ×10^3^/µL	9.70 [7.65–13.30]	9.80 [7.30–13.70]	11.20 [7.95–13.60]	0.71
Lymphocyte count, /µL	890.0 [648.0–1116.0]	856.0 [702.0–1564.0]	799.0 [573.0–1006.5]	0.37
Platelet count, ×10^4^/µL	14.70 [10.85–18.25]	12.30 [9.20–17.40]	14.30 [10.30–23.15]	0.48
Total bilirubin, mg/dL	0.80 [0.60–1.25]	1.00 [0.70–1.50]	0.80 [0.50–1.30]	0.58
Creatinine, mg/dL	0.730 [0.590–0.920]	0.760 [0.580–1.050]	0.690 [0.535–0.850]	0.14
Total protein, g/dL	5.10 [4.70–5.95]	5.02 [4.70–5.70]	5.00 [4.50–5.50]	0.99
Albumin, g/dL	2.75 [2.30–3.10]	2.70 [2.40–2.80]	2.30 [2.05–2.70]	0.01
Total cholesterol, mg/dL	139.5 [112.0–168.0]	137.0 [125.0–155.5]	128.5 [110.8–159.3]	0.27
CRP, mg/dL	9.21 [5.10–13.8]	7.45 [5.31–12.32]	9.75 [5.55–15.6]	0.62
Zinc, µg/dL	46.0 [37.0–59.0]	41.0 [30.0–47.0]	34.0 [27.0–42.5]	<0.01

**Table 3:** Primary and secondary endpoints according to daily zinc dosage.

	<15 mg/day	15–50 mg/day	>50 mg/day	*p*-Value
Primary Endpoint				
30-day survival, % (95% CI)	90.7 (84.5–96.9)	100.0 (N/A)	94.7 (90.1–99.3)	0.30
HR for 30-day mortality (95% CI)	1 (reference)	N/A	0.560 (0.183–1.712)	0.31
Secondary Endpoints				
90-day survival, % (95% CI)	88.3 (81.0–95.6)	85.6 (66.9–100.0)	86.2 (77.9–94.5)	0.97
ICU length of stay, median [IQR], days	14 [10–18]	16 [10–21]	16 [11–19]	0.35
Hospital-acquired pneumonia, % (95% CI)	54.7 (44.2–64.8)	31.3 (14.2–55.6)	42.6 (33.0–52.7)	0.11
Serum zinc concentration, median [IQR], µg/dL				
Week 2	63.5 [53.8–75.3]	69.0 [51.0–88.0]	60.0 [46.8–79.0]	0.37
Week 3	81.0 [69.0–94.0]	82.0 [68.0–101.0]	82.5 [64.0–105.0]	0.95

**Table 4:** Comparison of baseline characteristics and serum zinc concentrations between survivors and non-survivors.

	Survival (N = 183)	Death (N = 13)	*p*-Value
Age, median [IQR], year	61.0 [42.0–74.0]	74.0 [63.0–78.0]	0.076 *
Male sex, No. (%)	135 (73.8)	5 (38.5)	0.011 **
BMI, median [IQR]	24.8 [22.1–26.9]	24.9 [21.1–28.2]	0.881 *
Hospital-acquired pneumonia, No. (%)	86 (47.0)	6 (46.2)	1.000 **
ISS, median [IQR]	29.0 [25.0–38.0]	25.0 [25.0–36.0]	0.990 *
APACHE II score, median [IQR]	26.0 [22.0–30.5]	28.0 [23.0–31.0]	0.241 *
CONUT score, median [IQR]	9.0 [6.0–10.0]	7.0 [5.0–10.5]	0.639 *
Renal replacement therapy, No. (%)	6 (3.3)	1 (7.7)	0.386 **
Serum zinc concentration, median [IQR], µg/dL			
Upon admission	41.0 [32.0–50.0]	43.0 [32.0–61.0]	0.748 *
Week 2	63.0 [51.0–78.0]	58.0 [55.0–60.0]	0.240 *
Week 3	82.5 [67.2–101.0]	79.0 [60.5–93.5]	0.734 *
Laboratory findings upon admission [IQR]			
White blood cell count, ×10^3^/µL	10.2 [7.7–13.6]	9.1 [8.5–13.2]	0.581 *
Lymphocyte count, /µL	846.0 [644.0–1056.0]	733.0 [490.0–1033.0]	0.487 *
Platelet count, ×10^4^/µL	14.6 [10.7–21.0]	11.2 [5.7–15.2]	0.043 *
Total bilirubin, mg/dL	0.8 [0.6–1.3]	1.0 [0.6–1.3]	0.545 *
Creatinine, mg/dL	0.7 [0.6–0.9]	0.7 [0.5–0.9]	0.847 *
Total protein, g/dL	5.0 [4.6–5.7]	5.2 [4.7–6.0]	0.494 *
Albumin, g/dL	2.6 [2.1–3.0]	2.4 [2.4–2.8]	0.919*
Total cholesterol, mg/dL	134.0 [112.0–161.5]	167.0 [121.5–206.0]	0.135 *
CRP, mg/dL	9.0 [5.1–14.5]	13.7 [7.8–16.9]	0.154 *

* Mann–Whitney *U* test, ** Fisher’s exact test.

**Table 5:** Multivariate logistic regression analysis of the effect of zinc supplementation on 30-day survival.

Variables	Adjusted Odds Ratio (aOR)	95% CI	*p*-Value
Zinc Supplementation Dose			
≤50 mg/day (reference)	1	-	-
>50 mg/day	0.89	0.25–3.24	0.86
Age (per 1-year increase)	1.03	0.99–1.07	0.11
CONUT score (per 1-point increase)	0.86	0.69–1.09	0.21

**Table 6:** Multivariate logistic regression analysis of the effect of zinc supplementation on 90-day survival.

Variables	Adjusted Odds Ratio (aOR)	95% CI	*p*-Value
Zinc Supplementation Dose			
≤50 mg/day (reference)	1	-	-
>50 mg/day	1.11	0.40–3.05	0.845
Age (per 1-year increase)	1.04	1.01–1.07	0.019
CONUT score (per 1-point increase)	0.95	0.78–1.14	0.566

**Table 7:** Multivariate logistic regression analysis of the effect of zinc supplementation on hospital-acquired pneumonia.

Variables	Adjusted Odds Ratio (aOR)	95% CI	*p*-Value
Zinc Supplementation Dose			
≤50 mg/day (reference)	1	-	-
>50 mg/day	0.64	0.34–1.22	0.179
Age (per 1-year increase)	1.03	1.01–1.05	<0.001
CONUT score (per 1-point increase)	1.12	0.99–1.27	0.07

## Data Availability

The data presented in this study are available on request from the corresponding author due to privacy restrictions.

## Abbreviations

The following abbreviations are used in this manuscript:

ICU	Intensive care unit
EICU	Emergency and critical care intensive care unit
ISS	Injury Severity Score
APACHE II	Acute Physiology and Chronic Health Evaluation II
CONUT	Controlling Nutritional Status
ESPEN	European Society for Clinical Nutrition and Metabolism
WBC	White blood cell
CRP	C-reactive protein
CI	Confidence interval
HR	Hazard ratio
